# Tannerism

**Published:** 1880-08

**Authors:** 


### Editorial.

### TANNERISM.

Well, Dr. Tanner still lives, and the world believes he fasted forty days. Has our nature changed? Can man overcome his physiological wants by dint of mental exertion, or has an imposition been practiced? Time is required for the solution of the problem. Experience is contradicted in the uniformity of the pulsations and respiration for such an unusually extended length of time. Half a week has been considered a sufficient time to induce irritability of the heart sufficient to cause over a hundred pulsations, while in the experiment seventy-four seem to have been the usual number up to the expiration of six weeks.

Physiologists must turn back, if they think the test a fair one, and seek the cause of this wonderful phenomenon in the case of Tanner, or ferret out the point of departure from truth in their former investigations.

We would not be set down as skeptical on this recent exhibition, nor as an unbeliever in what was taken as established truths in science previously. We shall therefore wait patiently for further evidences, pro and con, before taking position on the subject. Better never form an opinion than after doing so, find it necessary to change.

If, however, we should be required to decide upon the matter with the lights present, the decision would be adverse to the fairness of the test. The brain, organs of respiration and circulation, could not, it seems to us, have performed their functions so perfectly without nourishment.

